# Supplementary material for: The use of ultraviolet light generated from light-emitting diodes for the disinfection of transvaginal ultrasound probes
Source: PLoS One. 2024 Feb 23;19(2):e0298449. doi: 10.1371/journal.pone.0298449 (PMC10890779; doi:10.1371/journal.pone.0298449)
Supplement: Attachment — Submitted filename: Response to Reviewers.docx [file pone.0298449.s001.docx]

Amitava Mukherjee

Academic Editor

Plos One

2^nd^ January 2024

RE: The use of ultraviolet light generated from light-emitting diodes for the disinfection of transvaginal ultrasound probes

Dear Editor,

We have read the reviewer comments and responded to those one by one below. All changes incorporated in the revised manuscript are highlighted red with track changes.

**Reviewer 1:**

Reviewer #1: The results of the studies “The use of ultraviolet light generated from light-emitting diodes for the disinfection of transvaginal ultrasound probes” are predictable. UVC radiation is effective in removing microorganisms. The presented conclusions are of practical importance, therefore I propose to improve some aspects of the manuscript.

Reply: Thank you for appreciation our work. We have addressed the reviewer comment comments and added the information’s in the revised manuscript.

**Comments: 1. Line 187, 244, 298, 301, 302 What is the purity of reagents? Where do the reagents come from? The reagents must be described in sufficient detail.**

Reply: 1. We have provided purity (where applicable) and the source of each reagent in detail, please see in the revised manuscript:

1. **Page # 7, Line # 187-189:** autoclaved sterile phosphate buffered saline (PBS; 8 g NaCl, Ajax Finchem, AUS; 0.2 g KCl, Ajax Finchem, AUS; 1.4 g Na_2_HPO_4,_ Chem Supply, AUS; 0.24 g KH_2_PO_4,_ Chem Supply, AUS; purity of each ≥ 99 %, in 1000 mL of milli Q water).
2. **Page # 10, Line # 245-246:** (Oxoid, Basingstoke, UK) supplemented with MnSO_4_.H_2_O (500 ppm, Sigma-Aldrich, MO, USA, purity ≥ 99 %).
3. **Page # 12, Line # 298-303:** (BSA; Bovovgen Biologicals, Vic, AUS, purity ≥ 99%) and washed horse red blood cells (RBCs; Bovovgen Biologicals, Vic, AUS, purity ≥ 99% ), in two different combinations: Clean Condition with BSA (0.3 g ) in 100 mL of diluent (Tryptone 1 g; Basingstoke, UK; sodium chloride 8.5 g, Ajax Finchem, AUS; purity of each ≥ 99% in 1000 mL of water) and Dirty Condition with 3 g of BSA (Bovovgen Biologicals, Vic, AUS)
4. **Page # 12, Line # 298-303:** (magnesium chloride19.48 g; Chem supply, SA, AUS and sodium bicarbonate 35.02 g; Sigma, St, Louis, USA, purity of each ≥ 99 %, in 1000 mL of water)

**Comments: 2. How many repetitions of the experimental measurements were performed?**

Reply: Each experiment was in performed in duplicate. Please see in the revised manuscript **Page # 13, Line # 315.**

**Comments: 3. Give the name and version of any software used, e.g. in Statistical Analysis**

Reply: Graph pad prism 8 version 8.0.2 was used for statical analysis and written in revised manuscript **(Page # 13, line# 307-308).**

**Comments: 4. I recommend adding Bacteria Detection Images to the manuscript. This visualization will complement the study.**

Reply: Thank you for your suggestions. We have included following figure in the manuscript. **(Page # 14).**

**A**

**B**

**C**

**D**

**E**

**F**

Figure 3. Detection of bacteria on chocolate blood agar. Figure 3A-C represent the bacterial colonies on the plate of non-UVC treated probe while Figure 3D-F represent the bacterial colonies on the plates of UVC treated probe.

**Comments: 5. Some samples are missing from the experiment description, e.g. Sample 2, 3, 4, .... What does this mean? Are these reference tests?**

Reply: We apologies for inconvenience. We have clarified this in the revised manuscript by adding the information’s in the Table legend.

Please see **Page # 13, line# 307-308.** “The table only includes sequentially listed sample numbers (1-30) that exhibited bacterial growth while those sample numbers were omitted where bacterial growth was absent”.
